# A Peer-Led School-Based Intervention for Safer Internet Use Among Primary School Students: A Controlled Pre–Post Study

**DOI:** 10.7759/cureus.105302

**Published:** 2026-03-16

**Authors:** Evangelia Siafarika, Sotiria Makaroni, Chara Tzavara, Eleni Panagouli, Loretta Thomaidou, Artemis Tsitsika, Maria Tsolia

**Affiliations:** 1 Second Department of Pediatrics, National and Kapodistrian University of Athens (NKUA) Panagiotis and Aglaia Kyriakou Children's Hospital, Athens, GRC; 2 School of Medicine, National and Kapodistrian University of Athens (NKUA), Athens, GRC; 3 Department of Basic Sciences, Medical School, University of Nicosia, Athens, GRC

**Keywords:** gaming, internet, intervention, mobile phone, social networking

## Abstract

Introduction

Increasing evidence links problematic internet use and excessive digital engagement with psychosocial vulnerability among youth. While adolescent populations have been extensively studied, preventive interventions targeting late childhood remain limited. This study examined changes in adolescents’ internet-related behaviors - social networking time, device use frequency, gaming, and compulsive internet use (10-item Compulsive Internet Use Scale (CIUS-10)) - associated with participation in a school-based intervention.

Methods

A controlled pre-post design was implemented among 235 fifth- and sixth-grade students (intervention group n = 125; control group n = 110). Twelve peer leaders were selected through voluntary participation and multi-criteria assessment and received structured training in digital literacy and peer facilitation. Behavioral indicators of internet use were assessed at baseline and two months after intervention. Normality of continuous variables was assessed using the Kolmogorov-Smirnov test. Descriptive statistics included means, standard deviations (SD), frequencies (N), and percentages (%), according to the type of variable. Between-group comparisons were performed using Mann-Whitney U (continuous variables), Chi-square or Fisher’s exact tests (categorical variables), and Wilcoxon signed-rank tests (pre-post comparisons groups).

Results

No baseline differences were observed between groups. Post-intervention analyses demonstrated statistically significant reductions in the intervention group in daily social networking time, device use frequency, online interaction behaviors, and gaming activities (p < .001; small to moderate effect sizes). No comparable pattern was observed in the control group.

Conclusions

Structured peer-led interventions may represent a feasible early preventive approach for promoting healthier digital engagement patterns in primary school settings.

## Introduction

The rapid digitalization of childhood has reshaped developmental environments. Children increasingly access internet-enabled devices at earlier ages, engaging in social networking, gaming, and online communication as part of their daily routines. While digital engagement offers educational and social opportunities, concerns have emerged regarding excessive or dysregulated use [1].

Problematic internet use (PIU) has been conceptualized within behavioral addiction frameworks and integrated into the Interaction of Person-Affect-Cognition-Execution (I-PACE) model [1], which posits that addictive behaviors arise from interactions between individual predispositions, affective responses, cognitive processes, and executive control mechanisms.

Meta-analytic evidence demonstrates significant associations between problematic internet use and depressive symptoms [2], problematic social media use and anxiety [3], and cybervictimization and depression [4,5]. Although much research focuses on adolescents, behavioral trajectories often begin in late childhood, suggesting that preventive interventions should target earlier developmental stages.

School-based prevention programs typically yield small-to-moderate effect sizes [6]. Peer-led models, grounded in social learning theory [7,8], leverage perceived similarity and peer influence mechanisms. During pre-adolescence, peer norms significantly shape behavior regulation.

Digital resilience frameworks further emphasize strengthening protective competencies and peer dynamics rather than focusing exclusively on restriction [9]. Despite growing interest, structured peer-led interventions targeting primary school digital behavior remain underexplored.

The objective of this study was to examine behavioral changes associated with participation in a school-based intervention among adolescents, focusing on key internet-related behaviors, including social networking time, device use frequency, gaming behaviors, and compulsive internet use tendencies assessed with the 10-item Compulsive Internet Use Scale (CIUS-10), using a non-randomized quasi-experimental design.

## Materials and methods

Study design

A controlled pre-post intervention design was employed to examine the association between participation in a structured peer-led school-based intervention and changes in digital engagement behaviors.

Classroom-level allocation to intervention and control groups was conducted based on administrative feasibility; randomization was not applied. Assessments were performed at two time points: pre-intervention (baseline) and two months following completion of the intervention (post-intervention). Given classroom-level allocation, potential clustering effects were considered; however, the limited number of participating classes did not permit multilevel modeling.

Participants

The total sample comprised 235 students enrolled in Grade 5 and Grade 6 of three public primary schools in the Attica region of Greece (intervention group: n = 125, control group: n = 110)*.* A total of nine classrooms participated in the study, including five classes assigned to the intervention group and four classes assigned to the control group. All students attending the selected classrooms and grades were eligible to participate. Student assent and written parental consent were required. 

No additional exclusion criteria were applied beyond absence during data collection. No attrition occurred between baseline and follow-up assessments. All available cases were included in the analyses (complete-case approach), as no missing data were recorded.

Peer leader selection procedure

Twelve students were selected as peer leaders using a structured multi-criteria process designed to identify students demonstrating pro-social orientation, emotional maturity, and communication capacity rather than popularity alone. Selection criteria included voluntary expression of interest, teacher evaluation of social responsibility and emotional maturity, classroom climate indicators, and multi-criteria review by the research team. This structured approach aimed to ensure balanced representation and suitability for peer facilitation roles.

Peer leader training and intervention structure

Peer leaders participated in structured workshops delivered by the research team before classroom implementation. Training components included: digital literacy education, safe and responsible internet practices, recognition of online risks, communication and facilitation skills, role-playing exercises, and supervised classroom activity rehearsal. Following training, the peer leaders implemented a structured classroom intervention consisting of four sessions delivered during regular school hours. Each session lasted approximately 45 minutes and was conducted within the classroom setting. The sessions were delivered across the five intervention classes and addressed topics including responsible internet use, digital habits, online communication behaviors, and awareness of online risks. All sessions were conducted under the supervision of a member of the research team, who was present in the classroom to monitor the implementation process, provide clarification when needed, and ensure that the educational content was delivered according to the intervention protocol. To ensure intervention fidelity, peer leaders followed a standardized intervention guide developed for the program. The guide specified the structure, activities, and key discussion points for each session, ensuring consistency in delivery across classrooms. The intervention duration was two months.

Measures

Students completed structured questionnaires assessing digital engagement patterns and behavioral indicators.

Digital engagement indicators

Measures included daily time spent on social networking platforms, frequency of device use (personal computer, laptop, mobile phone, gaming console, handheld devices), online social interaction behaviors (chat, messaging, social networking site participation, forum use, email), and online gaming activities.

Compulsive Internet Use Scale (CIUS-10)

Problematic internet use was assessed using the 10-item Compulsive Internet Use Scale (CIUS) [10,11]. The questionnaire is open access [11]. Items are rated on a 5-point Likert scale (0-4), with higher scores indicating greater compulsive internet use tendencies. In the present sample, internal consistency was satisfactory (Cronbach’s α > 0.80).

Statistical analysis

Statistical analyses were conducted using SPSS version 19.0 (IBM Corp., Armonk, USA). Normality of continuous variables was assessed using the Kolmogorov-Smirnov test. Descriptive statistics included means and standard deviations (SD) for continuous variables and frequencies (N) and percentages (%) for categorical variables.

Between-group comparisons at baseline were performed using Mann-Whitney U tests for continuous variables and Chi-square or Fisher’s exact tests for categorical variables. Within-group pre-post comparisons were conducted using Wilcoxon signed-rank tests.

Effect sizes for non-parametric analyses were calculated using the formula:

$\mathrm{r}=\frac{Z}{\sqrt[]{N}}$

Effect sizes were interpreted according to Cohen’s criteria (small: r = 0.10, moderate: r = 0.30, large: r = 0.50). The level of statistical significance was set at p < 0.05 (two-tailed).

Ethical considerations

The study was conducted in accordance with the ethical standards of the Medical School of the National and Kapodistrian University of Athens and the relevant educational authorities. Approval for implementation in participating schools was obtained from the competent educational bodies, including the Institute of Educational Policy (IEP), Athens (approval number Φ15/960/10022/Δ1). Written informed consent was obtained from parents or legal guardians of all participating students. Students provided verbal assent before participation. Participation was voluntary, and data were collected anonymously to ensure confidentiality.

## Results

Baseline characteristics

The baseline demographic characteristics of the study sample are presented in Table 1. No statistically significant differences were observed between the intervention and control groups regarding grade distribution (χ² = 0.002, p = 0.964), gender (χ² = 1.73, p = 0.187), body mass index category (χ² = 0.38, p = 0.535), country of birth (χ² = 1.32, p = 0.252), or family structure (Fisher’s exact test, p = 0.062). These findings indicate that the two groups were comparable at baseline.

**Table 1:** Baseline Characteristics The level of statistical significance was set at p < 0.05 (two-tailed).

Variable	Intervention (%)	Control (%)	p-value	Test statistic
Sixth grade	51.2	50.9	0.964	χ² = 0.002
Male	56.8	48.2	0.187	χ² = 1.73
Normal BMI	93.6	95.5	0.535	χ² = 0.38
Born in Greece	96.8	93.6	0.252	χ² = 1.32
Living with two parents	96.0	100	0.062	Fisher’s exact

Social networking time

The analysis of daily time spent on social networking sites showed a statistically significant reduction in the intervention group following the implementation of the peer-led program. Specifically, the mean scoredecreased from 4.61(SD = 2.65) before the intervention to 3.67 (SD = 1.83) after the intervention (Z = −4.52, p < 0.001). In contrast, no statistically significant change was observed in the control group, where the mean score slightly decreased from 4.57 (SD = 2.59) to 4.00 (SD = 2.62) (Z = −0.27, p = 0.788). These findings are presented in Table 2.

**Table 2:** Daily Time on Social Networking Sites The level of statistical significance was set at p < .05 (two-tailed).

Group	Pre-intervention Mean (SD)	Post-intervention Mean (SD)	Z	p-value	Effect	Effect size
Control group	4.57 (2.59)	4.00 (2.62)	-0.27	0.788	None (r=0.02)	Small: r = 0.10 Moderate: r = 0.30 Large: r = 0.50
Intervention group	4.61 (2.65)	3.67 (1.83)	-4.52	<0.001	Moderate (r=0.40)	

Device use frequency

Significant reductions in the frequency of device use were observed in the intervention group following the implementation of the intervention. Specifically, decreases were identified in the use of personal computers (45.6% vs 36.8%, Z = −3.89, p < 0.001), laptops (36.0% vs 28.0%, Z = −4.11, p < 0.001), mobile phones (42.4% vs 36.0%, Z = −3.62, p < 0.001), gaming consoles (38.4% vs 33.6%, Z = −3.74, p < 0.001), and handheld devices (69.6% vs 62.4%, Z = −3.28, p < 0.001). Effects ranged from small to moderate. No statistically significant changes were detected in the control group for any of the examined devices. Detailed results are summarized in Table 3.

**Table 3:** Changes in Device Use Frequency The level of statistical significance was set at p < 0.05 (two-tailed).

Device	Group	Pre (%)	Post (%)	Z	p-value	Effect	r	Effect size
Personal Computer	Intervention	45.6	36.8	-3.89	<0.001	Moderate	0.35	Small: r = 0.10, Moderate: r = 0.30, Large: r = 0.50
	Control	27.3	21.8	-0.49	0.621	Small	0.04	
Laptop	Intervention	36.0	28.0	-4.11	<0.001	Moderate	0.37	
	Control	12.7	13.7	-1.87	0.061	Small	0.17	
Mobile phone	Intervention	42.4	36.0	-3.62	<0.001	Moderate	0.32	
	Control	29.1	34.5	-1.26	0.209	Small	0.11	
Gaming console	Intervention	38.4	33.6	-3.74	<0.001	Moderate	0.33	
	Control	26.4	31.8	-1.14	0.254	Small	0.10	
Handheld devices	Intervention	69.6	62.4	-3.28	<0.001	Small	0.29	
	Control	62.7	53.6	-0.70	0.483	Small	0.06	

Online social interaction

Participation in several online communication activities significantly decreased in the intervention group after the implementation of the peer-led intervention. Specifically, reductions were observed in instant messaging (72.5% vs 61.6%, Z = −3.96, p < 0.001), social networking activities (64.9% vs 54.1%, Z = −3.75, p < 0.001), email communication (43.9% vs 34.4%, Z = −3.52, p < 0.001), and forum participation (29.2% vs 21.6%, Z = −3.61, p < 0.001). No statistically significant changes were observed in the control group for these activities. These findings are presented in Table 4.

**Table 4:** Online Social Interaction The level of statistical significance was set at p < .05 (two-tailed).

Activity	Group	Pre (%)	Post (%)	Z	p	Effect	r	Effect size
Instant messaging	Intervention	72.5	61.6	-3.96	<0.001	Moderate	0.35	Small: r = 0.10, Moderate: r = 0.30, Large: r = 0.50
	Control	71.9	71.2	-0.02	0.984	Small	0.00	
Social networking	Intervention	64.9	54.1	-3.75	<0.001	Moderate	0.34	
	Control	64.3	63.0	-1.93	0.053	Small	0.18	
Email communication	Intervention	43.9	34.4	-3.52	<0.001	Moderate	0.31	
	Control	44.1	43.5	-1.42	0.155	Small	0.13	
Forum participation	Intervention	29.2	21.6	-3.61	<0.001	Moderate	0.32	
	Control	29.9	29.2	-1.37	0.171	Small	0.12	

Online gaming

Similarly, the frequency of engagement in online gaming activities decreased significantly in the intervention group following the intervention. Significant reductions were observed for individual games (mean score: 3.71 vs 4.20, Z = −3.48, p < 0.001), shooter games (4.06 vs 4.49, Z = −3.51, p < 0.001), MMORP (massively multiplayer online role-playing) games (4.22 vs 4.58, Z = −3.43, p < 0.001), and strategy games (4.44 vs 4.69, Z = −3.29, p = 0.001). In contrast, no statistically significant changes were observed in the control group for most gaming activities. The results are presented in Table 5.

**Table 5:** Online Gaming Activities The level of statistical significance was set at p < 0.05 (two-tailed). MMORPG: massively multiplayer online role-playing game

Activity	Group	Pre Mean (SD)	Post Mean (SD)	Z	p	Effect	r	Effect size
Individual games	Intervention	3.71 (2.00)	4.20 (1.85)	-3.48	<0.001	Moderate	0.31	Small: r = 0.10, Moderate: r = 0.30, Large: r = 0.50
	Control	4.54 (1.76)	5.12 (1.25)	-1.15	0.249	Small	0.10	
Shooter games	Intervention	4.06 (2.01)	4.49 (1.67)	-3.51	<0.001	Moderate	0.31	
	Control	4.99 (1.63)	5.51 (1.06)	-3.27	0.001	Moderate	0.31	
MMORPG	Intervention	4.22 (1.90)	4.58 (1.58)	-3.43	<0.001	Moderate	0.31	
	Control	4.41 (1.77)	5.21 (1.42)	-0.98	0.327	Small	0.09	
Strategy games	Intervention	4.44 (1.80)	4.69 (1.65)	-3.29	0.001	Small	0.29	
	Control	5.06 (1.36)	5.04 (1.50)	-1.05	0.294	Small	0.09	

Overall findings

Overall, the peer-led intervention was associated with statistically significant reductions in several indicators of digital media use and online activities among students in the intervention group. In contrast, no comparable changes were observed in the control group. Effect sizes ranged from r=0.29 to r=0.40, indicating small to moderate intervention effects. These findings suggest that the peer-led educational program may contribute to healthier patterns of digital media engagement among preadolescent students.

## Discussion

The findings indicate consistent reductions in digital engagement patterns among students exposed to the peer-led intervention. Effect sizes were small to moderate, consistent with universal preventive interventions [6]. The peer-led model, grounded in social learning theory [7,8], may enhance norm internalization through perceived similarity. People’s digital behavior is influenced by their peers, and interactions with others may lead them to adjust how they act online [9]. Although mental health outcomes were not directly measured, reductions in digital exposure may align with literature linking excessive engagement to depressive symptoms and anxiety [2,3,4]. Within the I-PACE framework [1], early intervention may disrupt reinforcement cycles before consolidation into problematic patterns. Late childhood may represent a critical preventive window.

Limitations include a non-randomized design and short follow-up. Additionally, because the sessions were peer-led, students may have under-reported usage to align with the norms promoted by the intervention. Future randomized and longitudinal studies are recommended.

The present findings suggest that structured peer-led digital health interventions can be feasibly integrated within primary school curricula as part of broader health promotion frameworks. Evidence from systematic reviews indicates that peer-led school-based programs can produce meaningful, although typically modest, behavioral effects when implementation is structured and peer facilitators receive adequate training and supervision [6,12]. The current intervention followed this structured model, incorporating defined selection criteria, targeted training, and supervised classroom diffusion.

Importantly, the intervention emphasized reflective engagement and self-regulation rather than restrictive screen-time messaging alone. Contemporary digital competence frameworks support competence-building approaches that foster critical thinking, responsible engagement, and peer accountability [9]. Embedding peer-facilitated digital literacy modules within existing school health education programs may therefore enhance both feasibility and sustainability.

Given the minimal resource requirements associated with peer-led diffusion models, such approaches may represent cost-effective preventive strategies, particularly in large school populations.

At the policy level, early digital health promotion should extend beyond reactive responses to adolescent problematic internet use and incorporate preventive strategies during late childhood. Developmental models emphasize that digital behavior patterns consolidate progressively [1], suggesting that preventive ecosystems should be implemented early. 

Integration of structured peer-led digital health programs into national school health strategies could complement broader digital literacy initiatives and align with international preventive health promotion models. Interventions delivered before adolescence may alter behavioral trajectories.

Educational policy frameworks increasingly advocate for digital competence and resilience development rather than purely restrictive regulatory approaches [9]. Peer-led structures may provide a scalable mechanism for operationalizing such frameworks within school systems. Importantly, such integration should maintain structured training protocols and adult supervision to ensure intervention fidelity [6].

Future research should prioritize randomized controlled designs to establish causal inference and evaluate the robustness of observed effects. Longer follow-up periods are necessary to determine whether early peer-led interventions influence developmental trajectories of problematic digital engagement into adolescence.

Within the I-PACE framework [1], future studies may examine whether peer-led interventions moderate affective and cognitive reinforcement mechanisms associated with repetitive digital behaviors. Inclusion of validated measures of digital resilience, emotional regulation, and psychosocial adjustment would clarify underlying mechanisms of change.

Moreover, incorporation of objective digital usage indicators (e.g., device-recorded screen time) alongside self-report measures could enhance methodological rigor. Cross-cultural replication studies would strengthen generalizability and inform context-sensitive adaptation of peer-led digital prevention models.

## Conclusions

Structured peer-led school-based interventions appear to represent a feasible and developmentally appropriate strategy for promoting healthier digital engagement patterns among primary school students. Although the observed effects were generally small to moderate, they align with outcomes typically reported in universal school-based prevention programs. Implementing such interventions during late childhood may be particularly valuable, as digital habits and behavioral patterns are still developing at this stage. The present findings suggest that involving trained peer facilitators may contribute to modest but consistent reductions in several forms of online activity, including social networking, device use, and gaming behaviors.

Overall, integrating structured digital health initiatives into primary school settings may support early preventive efforts aimed at fostering balanced and responsible technology use. Future research employing randomized designs and longer follow-up periods is warranted to examine the durability of these effects and to further clarify the mechanisms through which peer-led approaches influence children’s digital behaviors.
